# Ozone Therapy as Adjuvant for Cancer Treatment: Is Further Research Warranted?

**DOI:** 10.1155/2018/7931849

**Published:** 2018-09-09

**Authors:** Bernardino Clavo, Norberto Santana-Rodríguez, Pedro Llontop, Dominga Gutiérrez, Gerardo Suárez, Laura López, Gloria Rovira, Gregorio Martínez-Sánchez, Esteban González, Ignacio J. Jorge, Carmen Perera, Jesús Blanco, Francisco Rodríguez-Esparragón

**Affiliations:** ^1^Research Unit, Dr. Negrín University Hospital, Las Palmas, Spain; ^2^Radiation Oncology Department, Dr. Negrín University Hospital, Las Palmas, Spain; ^3^Chronic Pain Unit of the Dr. Negrín University Hospital, Las Palmas, Spain; ^4^Instituto Universitario de Investigaciones Biomédicas y Sanitarias (IUIBS), Grupo BIOPHARM, Universidad de Las Palmas de Gran Canaria, Las Palmas, Spain; ^5^Grupo de Investigación Clínica en Oncología Radioterápica (GICOR), Madrid, Spain; ^6^Section of Thoracic Surgery, Department of Surgery, King Faisal Specialist Hospital and Research Center, Riyadh, Saudi Arabia; ^7^Experimental Medicine and Surgery Unit of Hospital Gregorio Marañón and the Health Research Institute of Hospital Gregorio Marañón (IiSGM), Madrid, Spain; ^8^Servicio Atención Especializada, Dirección General de Programas Asistenciales, Servicio Canario de Salud, Las Palmas, Spain; ^9^Unidad de Ozonoterapia, Hospital Quirónsalud, Barcelona, Spain; ^10^University of Saint George, Italy; ^11^Clinicanaria Internacional, Las Palmas, Spain; ^12^Department of Nuclear Medicine, DIMEC Center, Clínica San Roque, Las Palmas, Spain

## Abstract

**Introduction:**

This article provides an overview of the potential use of ozone as an adjuvant during cancer treatment.

**Methods:**

We summarize the findings of the most relevant publications focused on this goal, and we include our related clinical experience.

**Results:**

Over several decades, prestigious journals have published* in vitro* studies on the capacity of ozone to induce direct damage on tumor cells and, as well, to enhance the effects of radiotherapy and chemotherapy. Indirect effects have been demonstrated in animal models: immune modulation by ozone alone and sensitizing effect of radiotherapy by concurrent ozone administration. The effects of ozone in modifying hemoglobin dissociation curve, 2,3-diphosphoglycerate levels, locoregional blood flow, and tumor hypoxia provide additional support for potential beneficial effects during cancer treatment. Unfortunately, only a few clinical studies are available. Finally, we describe some works and our experience supporting the potential role of local ozone therapy in treating delayed healing after tumor resection, to avoid delays in commencing radiotherapy and chemotherapy.

**Conclusions:**

* In vitro* and animal studies, as well as isolated clinical reports, suggest the potential role of ozone as an adjuvant during radiotherapy and/or chemotherapy. However, further research, such as randomized clinical trials, is required to demonstrate its potential usefulness as an adjuvant therapeutic tool.

## 1. Introduction

Over several decades, prestigious journals have published articles on the capacity of ozone to induce direct damage on tumor cells and, as well, to enhance the effects of radiotherapy (RT) and chemotherapy (CT). Hence, many clinicians advocate its use in cancer treatment. However, these studies have been conducted* in vitro* in the laboratory and in some animal models. As such, the effects of ozone on tumor cells have been demonstrated in very different conditions from those employed in clinical ozone therapy (O_3_T) sessions. In clinical practice, usually the ozone does not enter into direct contact with the tumor cells; i.e., the ozone does not exercise a direct effect; its multiple effects are mediated by secondary messengers (such as H_2_O_2_ and 4-hydroxynonenal) [1, 2]. Apart from this, indirect mechanism-of-action ozone stimulates adaptive mechanisms that can induce modulations in the organism by affecting the immune system, blood flow and oxygenation, and oxidative stress. These indirect effects can be potentially beneficial in anticancer therapy, as has been suggested by some studies. However, the real value of ozone as an adjuvant in oncology can only be established by conducting clinical trials specifically directed towards specific tumors, and in well-defined circumstances such as those addressing tumor status and characteristics of the patients.

The objective of the present article is to revise the most relevant publications (mainly identified in PubMed) that propose the potential use of ozone as adjuvant during cancer treatment. Such insights would merit further research, including specific randomized clinical trials.

## 2. *In Vitro* Studies

For about 6 decades, the journal Nature has been publishing articles related to the effects of ozone and of ionizing radiation. In 1958, an article described ozone as having “an effect on humans similar to that of radiation”. The effects of ozone and ionizing radiation involve the generation of reactive oxygen species (ROS) such as superoxide or hydroxyl radicals and singlet oxygen as well as free radicals (e.g., atoms, molecules, or ions that have an unpaired valence electron). Free radicals and ROS are chemically reactive compounds which induce oxidative stress and their effects are partially palliated by antioxidants [3]. Of note is that the model for this study involved the inhalation of ozone, a methodology that has been specifically prohibited in O_3_T by current clinical guidelines [4, 5]. Also of note was that, if administered concomitantly with X-ray therapy, the effect was synergistic [6]. Four years later in 1962, the same authors published another article demonstrating that ozone was capable of producing chromosome breakages in human cell cultures, similar to that produced by X-rays [7].

In 1980 another prestigious journal, Science, described how, as a function of concentration, ozone could selectively inhibit (in cell cultures) the growth of different human tumor cells (lung, breast, and uterus) without affecting nontumor cell lines [8]. In 1987, a work described a cytotoxic effect of ozone on three ovarian carcinoma cell lines. The study, however, did not show this effect in one endometrial carcinoma cell line [9]. In 1990, ozone was described as having a potentiating effect on 5-fluorouracil in breast cancer and colon cancer cell lines; the combined treatment showed efficacy in cell lines previously resistant to 5-fluorouracil [10]. In 2007, a direct effect of ozone was shown in neuroblastoma cell cultures, in which ozone further potentiated the effect of cisplatin and etoposide, but not gemcitabine [11]. More recently, ozone was described as having a direct cytotoxic effect in human colon cancer cells, and ozone boosted the effect of cisplatin and 5-fluorouracil [12].

Summarizing the above, ozone has been shown in cell cultures to have variable effects as a function of its concentration (similar to some drugs), a direct action on some types of tumors (but not all) and, in some cases, potentiating the direct actions of RT and various CT drugs (again, not all the CT drugs studied). Further, some of the studies mentioned above demonstrated that the potentiating effect of ozone on RT and CT was related to the intracellular production of ROS and free radicals. ROS are reported to be tumorigenic in their ability to increase cell proliferation, survival, and cellular migration. ROS can induce DNA damage leading to genetic lesions that initiate tumorigenicity and subsequent tumor progression. In contrast, ROS can also induce cellular senescence and cell death and, therefore, can produce an antitumor effect [13]. Indeed, the antitumor effect of RT and many CT drugs is mediated by the production of ROS and free radicals in tumor cells.

However, in clinical practice, the tumor cells are in the interior of the body, are distributed in 3 dimensions (unlike the layers of tumor cells in the laboratory), and infiltrate healthy tissue with which there are complex relationships that can modify tumor microenvironment and tumor behavior. As such, except for very special circumstances (very superficial and noninfiltrating tumors from skin or mucosa) it is not possible for the ozone to act directly on tumor cells.

When systemic O_3_T is performed (principally by autohemotherapy or by rectal insufflation), ozone does not enter into the blood circulation and it is not able to reach tumor cells. As such, its effects are “indirect”, i.e., being mediated by the formation of secondary messengers and inducing a further adaptive response from the body in a hormetic dose-response relationship. Ozone concentration and effects do not follow a linear relationship: very low concentrations could have no effect and very high concentrations can lead to contrary effects to those produced by lower/middle concentrations [2, 14]. 4-hydroxynonenal (4-HNE) and H_2_O_2_ are among the most relevant secondary messengers induced by ozone during lung toxicity following airway inhalation [15, 16], but also in the course of the induction of beneficial effects during medical application [1, 2]. H_2_O_2_ can enter the cytoplasm of mononuclear cells, activate tyrosine kinase, and phosphorylate the transcription factor NF-kB which can act as regulator of signal transduction and, as such, represents a crucial mediator of host defense and immune responses [1, 2]. The important role of the transcription factor “nuclear factor erythroid-derived 2” (Nrf2) induction by ozone in order to enhance the antioxidant systems has been described recently [17–19].

Later in this article, some* in vivo* studies are described that are closer to the current clinical approach and demonstrate the potential effects of ozone in cancer treatment.

## 3. Animal Models 

Compared to* in vitro* studies, animal models more closely resemble the clinical state. Of note is that some studies in experimental animal models have indicated that ozone itself can exert “indirect action” on the progression of some tumor types.

In 2008, two different preclinical studies in mice were published in the same article. In the first study, cells of Ehrlich ascitic tumor and sarcoma 37 tumor were implanted in the ocular plexus of mice. After implantation, the animals were treated with ozone via rectal insufflation over 12 sessions using different ozone concentrations. In both tumors, a significant decrease in the numbers of lung metastases was observed, with lower numbers of tumor cells per mice at higher ozone concentrations [21]. In the second preclinical study, varying ozone concentrations were applied intraperitoneally for 15 days. Twenty-four hours after the last ozone treatment, Lewis lung carcinoma cells were inoculated via subcutaneous route. Relative to the control group, all the ozone pretreated groups showed a delay in tumor volume increase and the kinetics of tumor development, with a trend to better results when lower ozone concentrations had been used. Additionally, at 16 days after tumor cell inoculation, all animals in the control group had tumor development, while in the ozone-treated groups there were animals without signs of tumor growth, even after 35 days [21].

Also in 2008, an article was published using a model of carcinoma metastases of squamous cells in rabbits. The results showed that tumor growth tended to be produced at the site of inoculation (usually the ears), together with lung metastases. The O_3_T was administered intraperitoneally, i.e., the route that is frequently employed in small animals as being an approximation to the intravenous route in humans. In the group receiving O_3_T, 7 of the 14 rabbits survived and, of them, all but one showed complete response (complete disappearance of the tumor). Conversely, in the sham group (no ozone gas administered), of the 13 rabbits in the study, 3 survived and only 2 of them showed complete disappearance of the tumor. The ozone did not enter into direct contact with the tumor cells and, as such, the action of the ozone must have had an “indirect effect”. In the second part of the same study, the authors did not observe the same outcomes when immunosuppressors had been administered. This would suggest that the effect was mediated by boosting/stimulating the immune system [22]. Four years later, the same authors described ozone as having induced the synthesis of prostacyclins at the systemic level [23]. Years earlier the antimetastases effect of prostacyclins had been described [24].

A subsequent study by the same research group showed the indirect antitumor effect of ozone more clearly [25]. Employing the same rabbit experimental model, the authors observed that the animals receiving intraperitoneal ozone had a greater and statistically significant percentage regression of the tumor, relative to the sham group. Also, the tumor regression was associated with a significant increase in the intratumor infiltration of CD3+ T lymphocytes. Further, when new rabbits with induced tumors had received leukocytes from peripheral blood from rabbits that had tumor regression previously, 60% of the new animals had tumor regression. Conversely, when leukocytes from rabbits that had tumor progression were injected into the new rabbits, no antitumor effect was observed. This study demonstrates that, at least in this model, the ozone could exercise an indirect antitumor effect via modulation of the immune system.

The action of O_3_T as having a potential “indirect effect” has been clinically confirmed in several studies by Bocci et al. since the 1990s. The studies demonstrate that ozone can modulate the production of various cytokines (such as interleukins and interferon) and, as such, modulate the activity of the immune system which is responsible for the defense of tumor cells [1, 2, 26].

It is evident that the immune system plays a primordial role in the defense of the organism against infection and against cancer. Thanks to some recent clinical trials, the role of immune modulation as an antitumor strategy has been clearly established. As a result, monoclonal antibodies targeting cytotoxic T lymphocyte-associated antigen 4 (CTLA4), the programmed death-1 receptor (PD-1), and its ligand (PD-L1) have been approved by the European Medicines Agency (EMA) and/or the Food and Drug Administration (FDA) for the treatment of several tumors, especially melanoma and non-small cell lung cancer [27].

Immune modulation produced by O_3_T is nonspecific. The mode of action differs in relation to the activity of different types of lymphocytes and on the production of different types of cytokines. The extent of activity depends on environment, functional status, and ozone concentration. Indeed, several years ago Bocci et al. proposed the hypotheses that low-medium ozone concentrations could upregulate cytokines produced by CD4+ TH1 lymphocytes enhancing TH1/TH2 ratio while higher ozone concentrations could decrease this ratio [1, 2, 28–30]. Additionally, there could be considerable clinical gain in combining monoclonal antibody therapy and O_3_T.

Another study in an animal model (tumor-bearing mouse model with rectal cancer) showed an antitumor effect of intratumor injection of ozonated water [31]. The effect was probably mediated by a local immunomodulation effect induced by ozone. However, this method of ozone administration does not have high clinical application.

In addition to the indirect effect on the tumor, ozone potentiation of CT and RT could be of higher clinical relevance. A few experimental studies in animals with induced cancers have evaluated the effect of O_3_T in combination with RT. The results have been promising.

In 1974 Hernuss et al., studying Walker carcinosarcoma of the rat, reported that RT combined with O_3_T produced significantly better outcomes than RT alone. Tumor remission was 39% in the RT + ozone group versus 0% in RT group without ozone. Indeed, 6 months later, 17% of the ozone-treated animals remained alive without recurrences or metastases [32].

Conversely, in 1976 the same journal published several studies by Grundner et al. which did not find a radiosensitizing effect of ozone administered after RT in animals with Ehrlich-ascite carcinoma cells [33, 34], despite the authors having previously observed an enhancing effect* in vitro *[35].

Later, in 2015, with the same Ehrlich-ascite tumor cell model, intraperitoneal ozone was described as being effective (administered alone or concurrently with RT) with respect to antiedema and antitumor effects and with longer survival times. The effects were ozone concentration dependent [36].

More recently, in 2018, another study from Turkey evaluated the impact of ozone alone and also when administered concurrently with RT in an experimental rat model of tongue cancer. The study described an antitumor effect as well as improvement in survival in the ozone group compared to the cancer-group without any treatment. Additionally, the most remarkable observation was that tumor response and survival rates were significantly higher in rats treated with RT + O_3_T compared to those treated with RT alone. The median survival rates were 49 and 3.5 days, respectively [37].

All the experimental studies described above would warrant more investigation of the use of ozone in combination with RT and CT.

## 4. Tumor Hypoxia/Ischemia Modification as a Potential Method for Enhancing the Effect of Radiotherapy and Chemotherapy

Tumor ischemia and tumor hypoxia are well-known adverse factors in cancer. They favor resistance to RT and CT as well as progression and development of metastases despite therapy [38]. An increase in blood flow in the tumor during treatment could potentially increase the local delivery of the CT drugs and, as such, result in a more effective CT. Similarly, an increase in tumor blood flow during RT could potentially increase local delivery of radiosensitizing drugs and oxygen, thus increasing the effectiveness of the RT. Hypoxic cells can become 3 times more resistant to RT than the well-oxygenated tumor cells [39], and small increases in oxygenation in hypoxic cells result in a remarkable enhancement of the effectiveness of RT. Similarly, hypoxia decreases the effectiveness of several chemotherapeutic drugs [40].

The technique of polarographic probes (electrodes that measure, in mmHg, the pressure of O_2_ -pO_2_- in tissues and in tumors) has demonstrated the adverse effect of tumor hypoxia in the survival of patients with sarcoma, tumors in the uterine cervix and head and neck [38, 41, 42]. Similarly, given the relationships between hypoxia and angiogenesis, a higher risk of metastases has been described in the more hypotoxic tumors [42, 43].

Using an Eppendorf® system of polarographic probes, our research group has described how, with only 3 sessions on alternate days (very much less than standard O_3_T), there can be a measurable increase in tumor oxygenation [44]. However, of note is that the effect was not consistent in all tumor tissues, and the increase in tumor oxygenation was inversely related to the baseline tumor oxygenation; i.e., tumor oxygenation was only improved in the most hypoxic tumors (which are the tumors where the activity should be more clinically relevant). This selective effect is different from (and potentially complementary to) the effect produced by other techniques to modify tumor hypoxia, such as by increasing arterial pO_2_ using hyperbaric chambers or carbogen breathing [45] or increasing regional and tumor blood flow using spinal cord stimulation [46].

Ozone does not increase arterial pO_2_, but it can increase tissue and tumor oxygenation by several mechanisms. Ozone increases 2,3-DPG (2,3-diphosphoglycerate) concentrations in the erythrocytes. This produces changes in the hemoglobin (Hb) dissociation curve, displacing the HbO_2_/Hb equilibrium to the right (HbO_2_ + 2,3-DPG → Hb - 2,3-DPG + O_2_). The increase can be measured in patients with diminished pretreatment baseline levels [47]. This effect on Hb could also combine with the Bohr effect (low affinity of Hb for O_2_ at lower pH). The end result is that Hb is displaced to the right and increases the delivery of O_2_ to the tissues.

Further, ozone can (1) improve the flexibility of erythrocytes membranes and the rheological properties of blood and diminish blood viscosity [48, 49]; (2) induce the production of nitric oxide by vascular endothelial cells and, thus, produce vasodilation at the microcirculation level. These two effects decrease peripheral vascular resistance, which, as a result, gives rise to an increase in blood flow according to Poiseuille's law [50].

Of note is that repeated sessions are required (Bocci et al. postulate a cycle of >15 sessions) for the existence of sufficient stimulus in the bone marrow such that the new erythrocytes formed would express the improved biochemical constituents induced by the ozone [2].

Our studies on the effects of ozone on blood flow agree with the above-mentioned observations of tumor oxygenation. In a related study, we also assessed the effect of ozone (after only 3 sessions on alternate days) on (1) common carotid artery blood flow (ml/min) using Doppler quantification and (2) diastolic velocity (cm/s) in the middle cerebral artery using transcranial Doppler. The blood flow parameters increased by 75% in common carotid artery and 33% in middle cerebral artery after the third session. Even without additional sessions, the improvements continued to be significantly higher 1 week later: 29% and 18%, respectively. These findings support the concept that the effect of O_3_T on blood flow can be long lasting [51].

Once more, the effects were not the same for all patients since the improvements observed were inversely related to the baseline status [51]. This effect is different from that observed using a different nonpharmacological technique of blood flow modification where we observed that cervical spinal cord stimulation induced a consistent effect in almost all patients; i.e., (1) all patients showed an increase in blood flow in common carotid artery (mean increase: 50%); (2) almost all patients showed an increase in diastolic velocity in middle cerebral artery (mean increase: 26%) [52]. However, although the effect of ozone therapy was similar in magnitude, there was no similar level of consistency; the effects were not systematically reproduced in all patients and they were inversely proportional to the baseline blood flow values, i.e., higher increase in those arteries with lower baseline values. Again, this effect of O_3_T was “patient-dependent” which is in accordance with the concept of a trend towards a regulatory effect. This inverse relationship with baseline status is the same as that we have commented upon with respect to tissue oxygenation in tumors [44]. These observations have also been described for oxygenation in anterior tibialis muscle [53] and for the modulation of 2,3-diphosphoglycerate levels [47]. These effects of an inverse relationship with baseline status suggest that ozone tends towards facilitating the capacity of autoregulation via a redistribution of blood flow from the tissues that are well-vascularized (or oxygenated) to those other tissues that are not.

**Figure 1:**
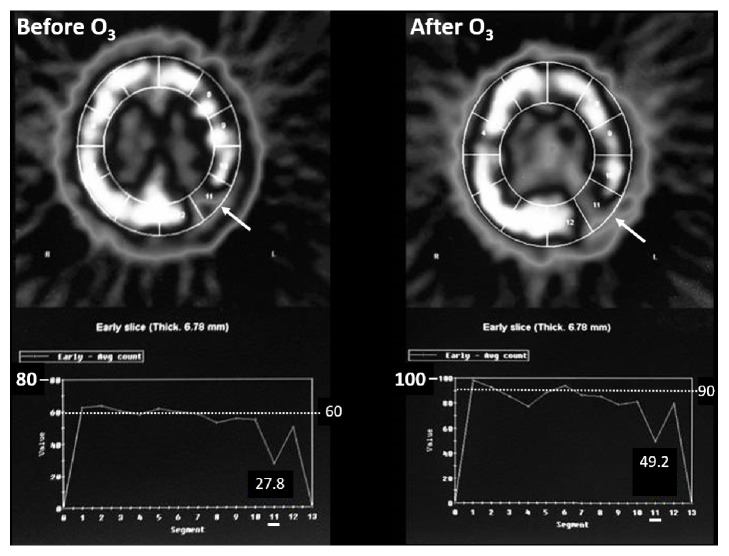
**Ozone therapy and cerebral blood flow assessed by SPECT-ECD. **Cerebral blood flow assessed by single photon emission computed tomography (SPECT) with ECD (^99m^Tc-ethyl cysteinate dimer); the tracer correlates with cerebral blood flow. The figure depicts a 68-year-old patient with a left parietooccipital glioblastoma (astrocytoma Grade IV) following subtotal resection. SPECT with ECD was carried out before (*Left*) and after 3 O_3_T sessions on alternate days (*Right*). After 3 sessions of O_3_T, (1) overall SPECT-index in brain increased from 60% to 90% and (2) in the tumor area (section #11) SPECT-index increased from 28% to 49%, increase >50%. Note that there are different scales before and after O_3_T.

Potentially, the effects described in common carotid artery and middle cerebral artery could also apply to tumor blood flow levels in ischemic tumors and could explain the observation of increase in pO_2_ in the most hypoxic tumors. This potential effect at regional/tumor blood flow level is supported by our observations using single photon emission computed tomography (SPECT). In a short study in patients with high-grade brain tumors, we assessed tissue blood flow in healthy brain and the tumor bed after O_3_T by autohemotherapy on 3 alternate days over one week (Figure 1).

Despite not being observed in all cases, in tumors in which O_3_T does increase tumor oxygenation, the effects of RT and CT could be enhanced, especially if this oxygenation increase is produced in zones of tumor hypoxia, i.e., in those that are most radio-resistant. Reviews, by Bocci et al. in 2005 [54] and, more recently, by Luongo et al. in 2017 [55], addressed the potential molecular and cellular pathways related to the effects of ozone on tumor hypoxia, tumor microenvironment, and tumor development [54, 55]. Their hypotheses are further supported by an experimental study of diabetic nephropathy in rats, which reported that ozone diminishes the previously elevated expression of hypoxia-inducible factor-1*α* (HIF-1*α*) [56]. Theoretically, more oxygenation (lower hypoxia) could inhibit HIF-1*α* activity in tumors, and this effect could reduce tumor-neoangiogenesis and further metastases. However, more studies are needed to address specifically whether an increase in O_2_ delivery to hypoxic tumors can downregulate HIF-1*α*.

To conclude this section, it is relevant to mention that the potential role of hypoxia modification during cancer treatment has been well described in two meta-analyses by Overgaard, which showed a clinically relevant impact on survival, especially in patients with head and neck tumors [57, 58]. It needs to be noted, however, that 15-30 minutes of high arterial pO_2_ levels produced by using hyperbaric chambers tends to produce an adverse regulatory vasoconstriction which leads to further tumor hypoxia [45]. Because of the potential effects on rheological parameters and blood flow, ozone could potentially decrease or delay vasoconstriction secondary to hyperoxia [59, 60]. As such, it would be valuable to explore the potential “complementary effect” of combining hyperoxia-based techniques (hyperbaric chambers and carbogen breathing) with O_3_T when applying RT and/or CT in the treatment of tumors.

## 5. Clinical Studies with O_3_T during RT and CT

Unfortunately, no randomized clinical trials (RCT) have been conducted, to date, to assess the effects of O_3_T in patients scheduled to receive traditional cancer treatment. Indeed, there are only few studies describing the use of ozone in combination with RT or CT. We will describe the most relevant findings in the few existing studies.

Several studies have been conducted with O_3_T at the University of Vienna. In 1974 an experimental study was published (already described, above) [32]. The same team also published a clinical study with 45 female patients with gynecological carcinoma treated with radiotherapy and ozone. The findings included a faster regression of pelvic tumors together with a decrease in radiation-induced side effects [61]. Interestingly, the authors stressed that the best results were achieved in patients treated because of recurrence of poorly oxygenated genital tumors. This is in accordance with our findings of a greater increase in pO_2_ in the most hypoxic tumors [44]. However, the next two studies had not been focused on ozone effects on the tumor. The studies in 40 women with gynecological cancer reported that, 10 minutes after ozone application, there were decreased levels of lecithin, lysolecithin, cephalin, sphingomyelin [62], fatty acids, and triglycerides [63]. In the latter study, 21 women with progressive cervical cancer (Stages III and IV) receiving RT with additional O_3_T showed a small decrease in IgG, IgA, and IgM, but the changes were not statistically significant [64].

In 1998 In Cuba, in was published a study in 70 patients with prostate cancer (Stages T1 and T2) treated with RT with and without concurrent rectal insufflation of ozone, the group of patients treated with RT + ozone showed greater and quicker decreases in the levels of PSA than the group of patients treated with RT alone [21].

Towards the end of the 1990s we conducted a comparative study in 19 patients with advanced head and neck cancer. We evaluated 2 groups: one with neoadjuvant CT (before commencing RT and concurrent 5-fluorouracil) and the other group without neoadjuvant CT but with O_3_T (by autohemotherapy method) during concurrent RT and 5-fluorouracil. Both patient groups had the same median survival (8 months) despite the fact that the group treated with ozone had included significantly higher numbers of patients with more adverse prognostic factors such as older age, larger size of metastatic cervical adenopathies, and higher percentage of patients with metastases at distance [65].

In 2003, Bocci et al. initiated an open study of ozone therapy in chemotherapy-resistant cancer patients [54]. Preliminary findings revealed that, in patients with a Karnofsky performance status of <40% (on a scale of 0 to 100% where 0 represents death and 100% reflects normal activity/function and no evidence of disease), no effect on disease progression was observed. Patients with a Karnofsky status of ≥70% reported an improvement in quality-of-life after 30-45 treatments, even those with diffused metastasis (usually liver or lungs). The lack of a control group and subjective nature of the outcome measure prevent definitive conclusions being reached, a point conceded by the authors [54].

Also in 2003, a Russian study reported the potential usefulness of adding parenteral ozone therapy to the standard treatment in 90 patients with hepatic dysfunction secondary to cancer-related obstructive jaundice. O_3_T facilitated a more rapid arrest of hepatic dysfunction and endogenous intoxication [66].

Between August 2005 and December 2008, we enrolled 7 patients with high-grade gliomas after surgery or tumor biopsy. The patients received O_3_T by autohemotherapy during the standard treatment of RT plus concurrent adjuvant temozolomide. Overall survival in the 6 patients with glioblastomas (Grade IV tumor) was similar to the standard treatment without ozone, including some long-term survivors. The patient with anaplastic astrocytoma (Grade III tumor) is alive after 11 years, with a good quality-of-life (Karnofsky 100%). However, only one patient cannot be considered representative. The aim of the study was to evaluate the potential changes in cerebral blood flow and the tumor bed (Figure 1). The study was closed early because of difficulties in recruitment.

In 2012, a report on 40 patients with advanced non-small cell lung cancer was communicated. The patients were treated with and without concomitant O_3_T by autohemotherapy (once a week for 12 weeks) and subcutaneous injection of* Viscum album fermentatum *(a species of mistletoe, a phytocompound used in northern Europe since Celtic times). Patients treated simultaneously with ozone and extracts of* Viscum album* showed a significantly better quality-of-life score measured by the quality-of-life questionnaire-core 30 (QLQ-C30). Additionally, this patient group showed a significant decrease in plasma values of reactive species metabolites and an increase in biological antioxidant potential [67].

There have been isolated reports presented at various scientific congresses of cases of good, or very good, outcomes using intraperitoneal O_3_T in patients with advanced cancer and peritoneal carcinomatosis. We have highlighted (above) the encouraging outcomes observed in animal models [22, 25]. However, we have not encountered clinical publications in PubMed using the intraperitoneal approach, only 2 limited references in journals that are not indexed: one in Spanish [68] and the other in English [69].

## 6. Avoiding Delays in Commencing RT and CT

Finally, in this section we describe a different approach whereby ozone could offer a potential benefit as adjuvant during cancer treatment.

Ostensibly healthy patients, or more often those who are more predisposed to delay in wound healing because of diabetes or local infections, sometimes present delay in healing following tumor resection surgery. RT and CT act, predominantly, on rapidly growing tumor cells but, as well as those responsible for wound healing and tissue repair. RT and CT can lengthen, or even impede, the process of healing and can result in local complications. As such, it is usual to wait until the healing process is complete before initiating these treatments. However, delay in initiating RT and CT can encourage the growth of tumor cells resulting in tumor progression or tumor relapse and decrease in the probability of cure.

In 1916-1917 during the World War I, Stoker published in the Lancet his findings in 79 patients receiving topical O_3_T in a military hospital. The patients had various war wounds and ulcerations, many of which had become infected (this was before the discovery of penicillin in 1928). In his articles, Stoker classified the results as satisfactory from the humanitarian, scientific, and social points of view [70, 71].

**Figure 2:**
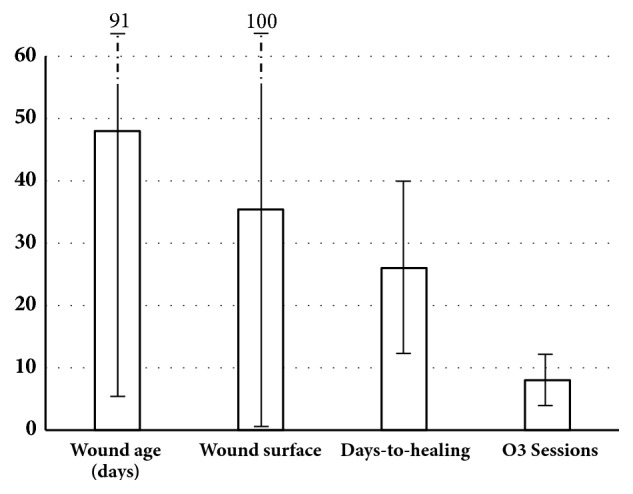
**Delayed healing in cancer patients. **Twenty-eight cancer patients treated with local O_3_T because of delayed healing after RT (3 patients) or after surgery (25 patients, 7 of whom previously received RT in the same anatomical area). Most patients needed further cancer treatment. The study group consisted of 18 females and 10 males, mean age 56±16 years (range: 21-95 years). Wound locations were 15 (54%) breast, 6 head and neck, and 7 in other areas. Mean duration of the wound: 48±43 days (range: 10-182 days), mean area of wound: 35.4±64.7 cm^2^ (range: 0.6-293 cm^2^), time-to-healing: 26±14 days (range: 4-50 days), ozone-sessions: 8±4 (range: 2-18). Local O_3_T was conducted at O_3_/O_2_ concentration between 50 *μ*g/ml and 20 *μ*g/ml, usually in two sessions per week. Reintervention for wound closure was programmed for 19 patients but was preempted in 16 of them (84%) due to O_3_T. Preliminary report was presented in 1999, in the X Congress of the Spanish Society of Radiation Oncology, and summarized the collaborative experience from the Hospital Quirónsalud (Barcelona) and the Dr. Negrín University Hospital (Las Palmas) [20]. Error bars show means ± SD.

**Figure 3:**
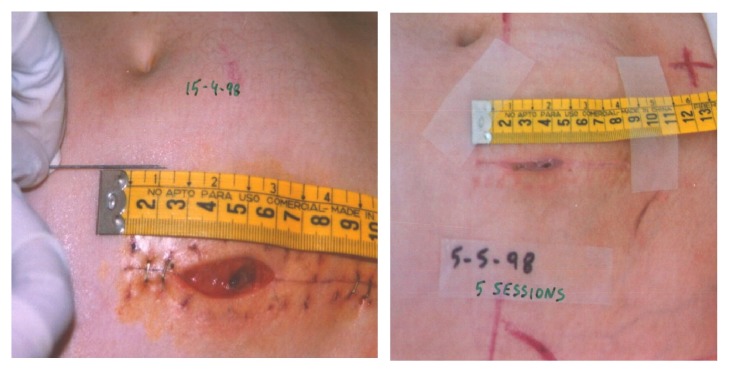
**Delayed healing after pelvic surgery. **Twenty-one-year-old patient with advanced and refractory Hodgkin lymphoma after several lines of CT and previous thoracic RT. A few days after commencement of pelvic RT, the treatment was discontinued because patient required surgery for appendicitis. (*Left*) Before O_3_T: wound delay 12 days after surgery and standard management (13.5 cc volume: 60x15 mm by 15 mm deep). We decided to apply local O_3_T and restart RT. (*Right*) After 5 O_3_T sessions during 3rd week of RT. Wound closed completely after 8 O_3_T sessions in 24 days during RT.

**Figure 4:**
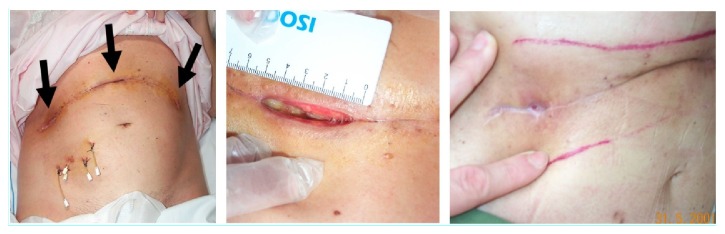
**Delayed healing after a pancreatic cancer surgery. **Fifty-three-year-old female patient with pancreatic carcinoma. During 1st surgery, tumor resection was not possible. She was treated with RT and CT. During a 2nd surgical intervention, complete tumor resection was not possible due to large vessel infiltration, and catheters were inserted for brachytherapy (a localized way for RT administration). (*Left*) Fourteen days after surgery. Note the catheters for brachytherapy in the lower-right abdomen. At this time, there were 3 wounds indicating delayed healing (arrows), all of which are larger than 40x10x10 mm. Pancreatic cancer cells were confirmed in the wounds. Local O_3_T was applied together with a 3rd course of RT (2nd external beam RT—this time, with electrons). (*Right*) Six weeks later. Complete wound healing (despite tumor cells) was observed during the 3rd RT after 15 sessions of local O_3_T.

There is considerable experience regarding the benefits of topical O_3_T (with or without systemic O_3_T) in the management of chronic ulcers secondary to infection [72, 73], vasculopathy [74], and diabetes [72, 75–77]. Apart from the above-mentioned effects favoring oxygenation and blood flow, ozone has been described as liberating various cytokines and growth factors and it can, on many occasions, cause a direct anti-infection effect, when applied locally [72, 73, 78]. Local ozone therapy (with or without systemic therapy), in patients with delayed healing following cancer surgery, can act locally to accelerate the process of healing and preempt, or at least decrease, the potential delay in initiating CT and/or RT. In 1999, at the X Congress of the Spanish Society of Radiation Oncology, we presented a preliminary report of the collaborative experience derived from the Hospital Quirónsalud (Barcelona) and the Dr. Negrín University Hospital (Las Palmas). The data represented 28 cancer patients with delayed healing after RT (3 patients) or after surgery (25 patients, 7 of whom had previously received RT in the same anatomical area) [20]. Almost all patients needed to continue with cancer treatment (RT and/or CT), and half of them were patients with breast cancer. Complete wound closure was achieved in 22 patients (79%) and improvement, but without complete resolution, in a further 4 patients (14%). The two patients without improvement included one patient with tumor progression a few weeks after treatment and another patient who developed a fistula in the airways and in whom the treatment could not have been properly carried out. Reintervention for wound closure was programed for 19 patients but in 16 of whom (84%) the reintervention was not necessary because of the O_3_T.  Figure 2 shows further data from this study, and some representative cases are described in Figures 3 and 4.

In the delayed healing before RT and/or before CT, the anatomical sites that benefitted most were those in which the local O_3_T could be applied without risk of airways involvement. Our experience has been with patients with delayed breast healing following surgical resection and those with abdominal interventions as well as those patients with head and neck tumors except those head and neck cancer patients with cutaneous larynx or pharynx fistulae.

Although we are not aware of published works focusing on the treatment of delayed wound healing in patients awaiting anticancer treatment, the application of ozonated water or ozonated oil represents a viable option. The oxidation products generated following the reactivity of ozone with fatty acids and other substrates can act as germicide and/or tissue restoration agents. The biological activities and stability of ozonized oils enable the development of standard formulations that deliver the benefits of ozone, with longer storage time (it remains stable for two years if kept refrigerated) [79]. The mechanism-of-action of ozonized oil in wound healing may be related, at least in part, to its antimicrobial effect and, as well, to its ability to activate local antioxidant mechanisms and promotion/liberation of growth factors and tissue reparation [79–84].

In patients with delayed healing following tumor resection, the local application of ozone, in conjunction with other standard treatments, could accelerate the healing process while avoiding potential delays in CT and/or RT commencement. This local approach is unlikely to produce any adverse systemic effect but could, instead, lead to optimized efficacy if further cancer-related treatment is required.

## 7. Final Considerations

From our experience, we recommend the following during cancer treatment:

- O3T should not be used as a substitute for any other oncological treatment (never as “alternative” medicine).
- O3T should always be “complementary” to conventional treatment (complementary medicine and/or integrative) in collaboration with the oncologists and other specialists responsible for the patient.
- Always have detailed and truthful information provided to the patient highlighting the studies that have suggested potential usefulness, but also that data from randomized clinical trials are lacking.
- Fully informed written consent must be obtained.

In general, therapeutic indications are established based on outcomes from clinical trials (randomized, whenever possible) but, unfortunately, these are difficult and costly to undertake, and more so when the treatment is nonpharmacological and, hence, lacking in support from the pharmaceutical industry [85]. However, RCTs are the most-approved route for studies of medications and therapies in the different clinical situations and, as such, RCTs need to be conducted to establish the potential clinical indications for O_3_T in various fields, including oncology.

The use of O_3_T as adjuvant for the management or prevention of cancer treatment toxicity has higher levels of scientific evidence and clinical justification. With this approach, O_3_T could be readily accepted because of its great potential in amplifying the administration of RT or CT in patients who may not be candidates because of their poor clinical status. This could include patients with poor renal, hepatic, pulmonary, and cardiac function, or when the option to apply the treatment is associated with high-risk toxicity. However, wider explanations of these topics would require a different review article.

To date, no RCTs have been conducted and so high level of evidence is lacking for the systematic use of O_3_T during anticancer treatment. However, many non-RCT reports do highlight the potential enhancement of RT and/or CT. Also, encouraging preclinical results have been described for intraperitoneal administration of ozone.

Therefore, as to the initial question of “ozone therapy as adjuvant for cancer treatment: is further research warranted?” we believe that the answer is an emphatic “yes”.
